# Supplementary material for: Assessing the reliability of a digital inclinometer app for measuring hip, knee, and ankle proprioception
Source: PLoS One. 2024 Sep 23;19(9):e0308737. doi: 10.1371/journal.pone.0308737 (PMC11419353; doi:10.1371/journal.pone.0308737)
Supplement: Attachment — Submitted filename: PONE-D-24-02242_reviewer.pdf [file pone.0308737.s002.pdf]

PLOS ONE

Assessing the Reliability of a Digital Inclinometer App for Measuring Hip, Knee, and Ankle Proprioception

--Manuscript Draft--

**Manuscript Number:**	PONE-D-24-02242
**Article Type:**	Research Article
**Full Title:**	Assessing the Reliability of a Digital Inclinometer App for Measuring Hip, Knee, and Ankle Proprioception
**Short Title:**	Hip, Knee, and Ankle Proprioception Reliability
**Corresponding Author:**	Ajit M.W. Chaudhari, Ph.D.<br>The Ohio State University<br>Columbus, OH UNITED STATES
**Keywords:**	proprioception; digital inclinometry; reliability; Knee; Hip; ankle
**Abstract:**	Proprioception can be defined as the ability of an individual to detect motion and position of the various joints in their bodies. Current tools for measuring proprioception lack consensus on their accuracy and validity; they also each have their own limitations, and, furthermore, present barriers to use for clinicians. We propose a new and reliable method for evaluating hip, knee, and ankle proprioception by utilizing a digital inclinometer app to measure joint position sense. The digital inclinometer app recorded the active joint position sense error after each of several trials per joint. To quantify the reliability of the digital inclinometer app, single-measurement and average-measurement intra-class correlation coefficients (ICC) were calculated for each joint's position sense error across trials. Both the hip (ICC(2,k) = 0.849) and knee joint (ICC(2,k) = 0.837) were found to have moderate to good reliability when the middle three of five trials were analyzed. Unlike the hip and knee, moderate to good reliability for ankle proprioception (ICC(2,k) = 0.785) was only achieved with the middle eight of ten trials. The results of this study indicate that this digital inclinometer app is able to accurately record joint position sense at the hip, knee, and ankle when the appropriate number of trials are collected; thus, allowing this tool and methodology to be considered for use in both clinical and research environments to measure proprioception, and furthermore, quantify proprioceptive deficits.
**Order of Authors:**	Sophia Chirumbole
	Rachel H Teater
	Megan Bals, DPT
	Daniel Richie, MS
	Scott M Monfort, PhD
	Ajit Chaudhari
**Additional Information:**	
**Question**	**Response**
**Financial Disclosure**<br><br>Enter a financial disclosure statement that describes the sources of funding for the work included in this submission. Review the submission guidelines for detailed requirements. View published research articles from *PLOS ONE* for specific examples.	The author(s) received no specific funding for this work.

Powered by Editorial Manager® and ProduXion Manager® from Aries Systems Corporation

This statement is required for submission and **will appear in the published article** if the submission is accepted. Please make sure it is accurate.<br><br>**Funded studies**<br>Enter a statement with the following details:<br>• Initials of the authors who received each award<br>• Grant numbers awarded to each author<br>• The full name of each funder<br>• URL of each funder website<br>• Did the sponsors or funders play any role in the study design, data collection and analysis, decision to publish, or preparation of the manuscript?<br><br>Did you receive funding for this work?	
**Competing Interests**<br><br>Use the instructions below to enter a competing interest statement for this submission. On behalf of all authors, disclose any competing interests that could be perceived to bias this work—acknowledging all financial support and any other relevant financial or non-financial competing interests.<br><br>This statement is required for submission and **will appear in the published article** if the submission is accepted. Please make sure it is accurate and that any funding sources listed in your Funding Information later in the submission form are also declared in your Financial Disclosure statement.<br><br>View published research articles from *PLOS ONE* for specific examples.	The authors have declared that no competing interests exist.

Powered by Editorial Manager® and ProduXion Manager® from Aries Systems Corporation

**NO authors have competing interests**<br><br>Enter: *The authors have declared that no competing interests exist.*<br><br>**Authors with competing interests**<br><br>Enter competing interest details beginning with this statement:<br><br>*I have read the journal's policy and the authors of this manuscript have the following competing interests: [insert competing interests here]*<br><br>* typeset	
**Ethics Statement**<br><br>Enter an ethics statement for this submission. This statement is required if the study involved:<br><br>• Human participants<br>• Human specimens or tissue<br>• Vertebrate animals or cephalopods<br>• Vertebrate embryos or tissues<br>• Field research<br><br>Write "N/A" if the submission does not require an ethics statement.<br><br>General guidance is provided below. Consult the submission guidelines for detailed instructions. **Make sure that all information entered here is included in the Methods section of the manuscript.**	All studies were approved by the Ohio State University Biomedical Institutional Review Board. Approval numbers include 2017H0149, 2017H0370, 2023H0145. Written consent was obtained for participants in 2017H0149, 2017H0370. 2023H0145 used anonymized data originally collected for non-research purposes.

**Format for specific study types**<br><br>**Human Subject Research (involving human participants and/or tissue)**<br>• Give the name of the institutional review board or ethics committee that approved the study<br>• Include the approval number and/or a statement indicating approval of this research<br>• Indicate the form of consent obtained (written/oral) or the reason that consent was not obtained (e.g. the data were analyzed anonymously)<br><br>**Animal Research (involving vertebrate animals, embryos or tissues)**<br>• Provide the name of the Institutional Animal Care and Use Committee (IACUC) or other relevant ethics board that reviewed the study protocol, and indicate whether they approved this research or granted a formal waiver of ethical approval<br>• Include an approval number if one was obtained<br>• If the study involved *non-human primates*, add *additional details* about animal welfare and steps taken to ameliorate suffering<br>• If anesthesia, euthanasia, or any kind of animal sacrifice is part of the study, include briefly which substances and/or methods were applied<br><br>**Field Research**<br><br>Include the following details if this study involves the collection of plant, animal, or other materials from a natural setting:<br>• Field permit number<br>• Name of the institution or relevant body that granted permission	
**Data Availability**<br><br>Authors are required to make all data underlying the findings described fully available, without restriction, and from the time of publication. PLOS allows rare exceptions to address legal and ethical concerns. See the PLOS Data Policy and FAQ for detailed information.	Yes - all data are fully available without restriction

A Data Availability Statement describing where the data can be found is required at submission. Your answers to this question constitute the Data Availability Statement and **will be published in the article**, if accepted.<br><br>**Important:** Stating 'data available on request from the author' is not sufficient. If your data are only available upon request, select 'No' for the first question and explain your exceptional situation in the text box.<br><br>Do the authors confirm that all data underlying the findings described in their manuscript are fully available without restriction?	
**Describe where the data may be found in full sentences. If you are copying our sample text, replace any instances of XXX with the appropriate details.**<br><br>• If the data are **held or will be held in a public repository**, include URLs, accession numbers or DOIs. If this information will only be available after acceptance, indicate this by ticking the box below. For example: *All XXX files are available from the XXX database (accession number(s) XXX, XXX.).*<br>• If the data are all contained **within the manuscript and/or Supporting Information files**, enter the following: *All relevant data are within the manuscript and its Supporting Information files.*<br>• If neither of these applies but you are able to provide **details of access elsewhere**, with or without limitations, please do so. For example:<br><br>*Data cannot be shared publicly because of [XXX]. Data are available from the XXX Institutional Data Access / Ethics Committee (contact via XXX) for researchers who meet the criteria for access to confidential data.*<br><br>*The data underlying the results presented in the study are available from (include the name of the third party*	All relevant data are within the manuscript and its Supporting Information files.

Powered by Editorial Manager® and ProduXion Manager® from Aries Systems Corporation

and contact information or URL).<br>• This text is appropriate if the data are owned by a third party and authors do not have permission to share the data.<br><br>* typeset	
Additional data availability information:	

Powered by Editorial Manager® and ProduXion Manager® from Aries Systems Corporation

Manuscript

Click here to access/download;Manuscript;Reliability_submission.docx

Assessing the Reliability of a Digital Inclinometer App for Measuring Hip, Knee, and Ankle Proprioception

Sophia G. Chirumbole[1], Rachel H. Teater[2], Megan M. Bals[3], Daniel R. Richie[4], Scott M. Monfort[5], Ajit M.W. Chaudhari[1,4,6]

[1]Mechanical & Aerospace Engineering, Ohio State University

[2]Mechanical Engineering, Vanderbilt University

[3]Physical Therapy, NovaCare Rehabilitation

[4]Biomedical Engineering, Ohio State University

[5]Mechanical & Industrial Engineering, Montana State University

[6]Health and Rehabilitation Sciences, Ohio State University

Abstract

Proprioception can be defined as the ability of an individual to detect motion and position of the various joints in their bodies. Current tools for measuring proprioception lack consensus on their accuracy and validity; they also each have their own limitations, and, furthermore, present barriers to use for clinicians. We propose a new and reliable method for evaluating hip, knee, and ankle proprioception by utilizing a digital inclinometer app to measure joint position sense. The digital inclinometer app recorded the active joint position sense error after each of several trials per joint. To quantify the reliability of the digital inclinometer app, single-measurement and average-measurement intra-class correlation coefficients (ICC) were calculated for each joint's position sense error across trials. Both the hip (ICC(2,k) = 0.849) and knee joint (ICC(2,k) = 0.837) were found to have moderate to good reliability when the middle three of five trials were analyzed. Unlike the hip and knee, moderate to good reliability for ankle proprioception (ICC(2,k) = 0.785) was only achieved with the middle eight of ten trials. The results of this study indicate that this digital inclinometer app is able to accurately record joint position sense at the hip, knee, and ankle when the appropriate number of trials are collected; thus, allowing this tool and methodology to be considered for use in both clinical and research environments to measure proprioception, and furthermore, quantify proprioceptive deficits.

Introduction

Proprioception involves the awareness of joint position and movement as well as the integration with other somatosensory, visual, and vestibular information to coordinate the individual's movement patterns [1]. Although there is no consensus among researchers to define proprioception, it can be generally described, as an "individual's ability to integrate the sensory signals from mechanoreceptors to thereby determine body segment positions and movements in space" [1]. One key function of human movement that depends on proprioception is postural control, as those with proprioceptive deficits often

have increased postural sway and decreased single leg balance [2–4]. Some researchers suggest deficits in proprioception correlate with increased risk for injury, decreased muscle strength and power, and long-term musculoskeletal disorders, such as osteoarthritis [2]. Although proprioception is presumed to be important to posture and movement, the literature in this area is limited, which is in part due to the lack of accepted standard methods for measuring it.

There are two common methods of measuring proprioception: threshold to detect passive motion (TDPM) and joint position sense (JPS) [5]. TDPM is measured by a tester passively moving a participant's joint in a direction (e.g., flexion, extension, etc.) and the participant then indicating when they sense movement of the joint. During JPS testing, the participant must remember and reproduce a target angle. There are two types of JPS testing, active and passive. Active joint position sense (aJPS) testing requires participants to remember a joint position angle and then attempt on their own to reproduce the joint angle. In passive joint position sense (pJPS) testing, the participant is still required to remember an angle, but the tester then moves the joint through the range of motion until the participant states they are at the remembered angle [6]. Unfortunately, TDPM and JPS methods of measuring proprioception are not directly comparable [7,8]. TDPM has been previously shown to be a more reliable measurement of proprioception, but it has less ecological validity compared to aJPS, as it depends solely on processing sensory feedback [1,8]. The aJPS method may be more functional and indicative of true proprioceptive accuracy [7,9] because it requires both sensorimotor integration and motor control [8]. In the current study, we chose to look at aJPS because functional proprioception during daily tasks always includes both sensorimotor and motor control components.

Evaluation of proprioception is important for rehabilitation, causing both clinicians and researchers alike to call for a new protocol to measure proprioception [7]. Proprioception is an increasingly important component of rehabilitation and has been shown to improve with targeted exercises [10]. Awareness of proprioceptive deficits is beneficial to clinicians to help focus interventions specific to patients' impairments and allow for optimal recovery. However, a lack of data exists about the

accuracy and validity of available tools [8,11]. Isokinetic dynamometers, such as the Biodex system, are commonly assumed to be a gold standard for many assessments of function including strength and proprioception testing [1]; however, this equipment is expensive, bulky, and not portable. Additionally, the straps, pads, and the inertia of the Biodex arm may provide additional somatosensory cues to the participant, which potentially make it a less ecologically valid assessment of joint proprioception. Electro-goniometers, potentiometers, inclinometers, and 2D and 3D motion analysis have all also been shown to take reliable joint angle measurements to determine proprioception [12–14]; however, these measurement tools require extensive data analysis and expensive equipment for interpretation [11]. These limitations can present clinicians with a barrier to measuring joint proprioception in populations that are at risk for having deficits. Recently, mobile apps have increasingly been investigated as means to quantify human movement for activity monitoring, functional assessment, and rehabilitation purposes [15]. However, the reliability and validity of these apps for assessing proprioception are currently unknown.

In the current article, three separate datasets were collected to establish the within-session reliability of app-based digital inclinometry for measuring aJPS. In addition to establishing within-session reliability, each joint dataset was analyzed separately for an additional secondary interest, or purpose. The secondary interest in performing the hip proprioception testing was to determine if conclusions can be drawn for both limbs from single limb hip aJPS error data (e.g. if one can gain information about the right limb, even if aJPS data is only collected on the left limb). Previously, the knee joint active repositioning angle error has been analyzed for between limb differences, but less so for the hip joint [16]. The secondary interest in performing the knee proprioception testing was to determine if knee aJPS error is consistent over time, more specifically, when trials are separated by an intervening activity, or a delay in time. This has been done previously by comparing data collected on separate days with the glenohumeral joint [17], and understanding the inter-session reliability of aJPS measurements is critical to use in clinical settings to track longitudinal changes. Lastly, the secondary interest in performing the ankle proprioception testing was to determine how many trials were needed to achieve acceptable reliability. In

current literature, there exists no gold standard for the appropriate number of trials to collect to achieve acceptable reliability of ankle JPS using a digital inclinometer app [18].

This article reports the reliability and validity of a digital inclinometer app that has been previously validated for quantifying pelvic motion [19]. Additionally, inter-limb and inter-session differences are explored in the hip and knee joints. Establishing the reliability and validity of app-based digital inclinometry for quantifying joint proprioception, including any inter-limb and inter-session significance, will enable researchers and clinicians to easily and cost effectively assess joint proprioception in individuals who may have proprioceptive deficits for a variety of patient populations.

Methods

Study participants

This article describes the analyses of three separate datasets collected to understand hip, knee, and ankle aJPS reliability. Deficits in memory can impede a participant's ability to recall the position independent of proprioceptive deficits and can confound data for this measurement [1]; therefore, only participants without any known cognitive impairment were included.

Hip proprioception

Forty-two participants were recruited and tested between the months of March and April 2017 at the Ohio State University. A total of 17 males and 25 females were included in this portion of the study, with ages ranging from 21-31 years old. Hip proprioception data were originally collected as part of an educational assignment for a course. Inclusion criteria included enrollment in a course on research methods for first-year Doctorate of Physical Therapy students, ability to fully bear weight on both lower extremities, no known cognitive impairments, and no known prior hip pathology. The institutional IRB approved subsequent use of these data for research.

Knee proprioception

Thirty healthy participants were recruited and tested between the months of August and December 2018 at the Ohio State University. A total of 19 males and 11 females were included in this portion of the study, with ages ranging from 22-27 years old. Inclusion criteria included no history of knee pain, no history of nerve damage in the lower extremities, no known cognitive impairments, and no previous knee surgery. All participants provided IRB-approved informed consent before participating.

Ankle proprioception

Forty-six healthy participants were recruited and tested in August of 2017 at the American Society of Biomechanics Annual Meeting in Boulder, Colorado. Demographic information, such as age and biological sex, was not recorded. Inclusion criteria included no known cognitive impairments and no history of nerve damage in the lower extremities. All participants provided IRB-approved informed consent before participating.

Instrumentation

This study used an iOS digital inclinometer app that measures an iPod's orientation (i.e., allowing us to measure the angle of one body segment relative to an earth-centered vertical or horizontal axis). All iPod models starting in 2011 have built-in triaxial accelerometers and gyroscopes along with software-based sensor fusion that allow them to estimate the orientation of the device as it moves in space [20]. Multiple trials were recorded at each joint to determine the digital inclinometer app's reliability. The CoreX Therapy app was used because it allows zeroing of the inclination at the starting position. The app reports both the largest deviation and the final deviation from starting position when recording is stopped and allows the use of the Play/Pause button on a wired or Bluetooth headphone or speaker to start and stop the measurement. This app also permitted unlimited storage of trials within the app for later recall and analysis. The CoreX Therapy app used Apple's built-in CoreMotion sensor fusion to estimate device

orientation with no additional low-pass filtering of the orientation angle. Participants were unable to view the screen of the iPod during testing so they could not use the app for any cues as to their position or position error.

Procedure

Hip proprioception

For all participants, a coin flip was used to randomize which limb would be tested first. The participants were blindfolded to remove visual information and allow for more accurate proprioception measurement, and headphones were placed in their ears to prevent them from hearing. Next, a belt holding an iPod with the digital inclinometer app installed was attached to the leg being tested, halfway between the anterior superior iliac spine and the patella (Fig 1). In one hand the participants held the button on the cord of their given pair of headphones, which were connected to the iPod running the app, and were instructed on how to use the headphone button to stop the app and record their data. Each participant was led through the experimental protocol (Fig 1). This process was repeated for a total of 5 trials on each leg.

Fig 1. Hip proprioception experimental protocol

Knee proprioception

For all participants, a coin flip was used to randomize which knee would be tested. Once the test limb had been established, it remained the test limb for the duration of the study. Participants were instructed to wear shorts to minimize external sensation from clothing.

For knee proprioception measurements, the iPod was secured to the participant's mid-tibia with a felt strap. A Bluetooth speaker connected to the iPod was handed to the participant so that they could reach the "play/pause" button, which was used to operate the test in the app. Each participant was asked

to stand on a short box placed directly in front of a wall with their non-tested leg to allow the tested foot to clear the floor. The tested extremity's anterior thigh was pressed against the vertical wall in front of them (to achieve vertical orientation of the thigh) with the knee fully extended (Fig 2). The participant wore a blindfold to remove visual information and allow for more accurate proprioception measurement.

Fig 2. Knee proprioception experimental protocol

Before initiating the first trial, the participant was taken through a step-by-step walkthrough of the test to ensure understanding of the protocol. After the walkthrough, each participant went through five trials of active range of motion within the experimental protocol (Fig 2). These five trials were recorded. Each participant then performed other tasks, including single-leg landings and isokinetic strength testing, for a few minutes before returning to repeat the entire protocol listed above for a second time (later referred to as delay).

Ankle proprioception

Participants were asked to remove the shoe from the foot being tested which was randomly chosen by a coin flip. Participants were instructed to wear socks and roll up their pants, if they cover their ankles, so that the ankle was clear and observable. Participants were instructed to lie supine on a table or treatment plinth. For ankle proprioception measurement, the iPod was secured to the participant's foot with a felt strap (Fig 3). iPod headphones were plugged into the iPod and handed to the participant so that they could reach the "play/pause" button, which was used to operate the test in the app.

Fig 3. Ankle proprioception experimental protocol

Before initiating the first trial, the participant was taken through a step-by-step walkthrough of the test to ensure understanding of the protocol. After the walkthrough, the participant went through a trial of active range of motion, following the experimental protocol (Fig 3). The participant's eyes were closed throughout the trials. This protocol was repeated nine more times, for a total of ten trials per participant.

Data and statistical analysis

For each joint, the aJPS error is reported as an absolute value, in degrees, from the start position. The interclass correlation coefficient (ICC) (2,1) reliability statistic was calculated for all recorded trials using SPSS Statistics software with a two-way random model and absolute agreement type (SPSS Statistics for Windows, Version 28.0. Armonk, NY: IBM Corp). The ICC (2,k) reliability statistic was calculated for the remaining data points after removing the minimum and maximum value for each testing session. The ICC (2,k) test applied to the remaining data points was used as the primary measure of test-retest reliability of the digital inclinometer app. Reliability was determined based on Portney & Watkins' recommendation that ICC values less than 0.5 are indicative of poor reliability, values between 0.5 and 0.75 indicate moderate reliability, values between 0.75 and 0.9 indicate good reliability, and values greater than 0.90 indicate excellent reliability [21].

To test our secondary hypotheses for each joint, further analyses were performed for both the hip and knee joints. In the hip joint, to explore consistency across limbs, the ICC (2,1) statistic was calculated for the "trimmed average" after removing the minimum and maximum aJPS error value for the testing session done on each limb. This ICC (2,1) test applied to the trimmed average was used as the measure of aJPS error consistency over the left and right limbs at the hip joint. As for the knee joint, to explore any differences over time, the ICC (2,1) statistic was calculated for the "trimmed average" after removing the minimum and maximum value for the testing session done before and after a delay in time, or intervening activity. This ICC (2,1) test applied to the trimmed average was used as the measure of aJPS error consistency over time at the knee joint.

Results

Hip proprioception

The hip aJPS error ranged between 0.1° and 10.6° from the learned position. The average error from the starting position was 2.4° with a standard deviation of 1.7°. Taking all five trials into account, the ICC (2,1) between trials was 0.388. After elimination of the largest two outliers within each participant, the ICC (2,k) was 0.849 (Table 1). The ICC (2,1) value represents poor reliability; however, the ICC (2,k) value represents good reliability. The inter-limb ICC (2,1) was found to be 0.508, which shows moderate agreement between limbs.

Table 1. ICC values demonstrating reliability of the proprioception measurement for each joint location.

	**Hip**	**Knee**	**Ankle**
**ICC (2,1)**	0.388 (5 trials)	0.276 (5 trials)	0.176 (10 trials)
**ICC (2,k)** (5 trials)	0.760	0.656	0.481[a]
**ICC (2,k)** (10 trials)			0.682
**ICC (2,k)** (middle 3 of 5 trials)	0.849	0.837	0.585[a]
**ICC (2,k)** (middle 8 of 10 trials)			0.785

[a]For the ankle, the ICC (2,k) of the 5 and middle 3 of 5 trials only used the first 5 trials collected.

Knee proprioception

The knee aJPS error ranged from 0° to 8.5°, with the average and standard deviation in error being 1.8° and 1.4°, respectively. Taking all five trials into account the ICC (2,1) between trials was 0.276 (Table 1). After elimination of the largest and smallest error values within each participant, the ICC (2,k) of the middle 3 of 5 trials was 0.837 (Table 1). Similarly to the hip proprioception data, the ICC (2,1) of the knee represents poor reliability; however, the ICC (2,k) of the knee represents moderate to good

reliability. When comparing between trials before and after the delay, the ICC (2,1) was 0.429. This value indicates poor repeatability between sessions.

Ankle proprioception

The ankle aJPS error ranged from 0° to 18.6°, with the average and standard deviation in error being 2.9° and 1.3°, respectively. Including all 10 trials, the ICC (2,1) was 0.176, indicating poor reliability for a single-measurement approach. After elimination of the highest and lowest value out of the first 5 trials collected for each participant, the ICC (2,k) was 0.585 (Table 1). This indicated that, unlike the hip and knee proprioception data, with only 5 trials the ICC (2,k) of the ankle demonstrated poor to moderate reliability. Increasing the number of trials to the middle 8 of 10 trials, the ICC (2,k) increased to 0.785, indicating moderate to good reliability.

Discussion

In this study, three separate datasets demonstrated that good within-session reliability can be achieved with active joint position sense error (aJPS) using app-based digital inclinometry. Both the hip and knee joints achieved good reliability with only five trials, where the ankle joint required up to ten trials to achieve similar reliability results. This could be due, in part, by the ankle's position being more distal than the other two joints studied, making the measurement of ankle proprioception more variable by nature.

This way of assessing proprioception reduces the clinical barriers mentioned in this article, such as expensive, bulky equipment and inefficient and complicated data analysis. Using a digital inclinometry app, as used in the current study, is simple to use, inexpensive, and easily portable. These are all qualities clinicians look for in a clinical tool, meaning this could be easily implemented in the clinical setting as a means of gaining valuable quantitative data on patient proprioception.

Hip proprioception

Results of the hip proprioception testing showed good test-retest reliability of the digital inclinometer app when recording five repeated trials. Results of the hip proprioception testing, analyzed between limbs, showed that there was moderate correlation in the joint position sense data. This suggests that one cannot assume that healthy individuals are symmetric in their hip proprioception.

These results build upon the results of a previous study, aiming to assess the validity and intra-rater reliability of a smartphone application called "Clinometer" for measuring hip, knee, and ankle sagittal ranges of motion, using a digital inclinometer as the reference standard [22]. In this study, Mohammad et al. found that the "Clinometer" app displayed excellent validity when compared to the digital inclinometer for hip movements. Additionally, the "Clinometer" app demonstrated excellent reliability for hip sagittal plane motion. Given that both Mohammad et al.'s assessment of range of motion and our measurement of joint position sense error require accurate measurements of segment positions relative to gravity, the current study's results support and build upon the results of this previous study by showing comparable reliability values, good reliability for joint position sense error at the hip, and moderate reliability when comparing between limbs.

Knee proprioception

Results of the knee proprioception testing showed good test-retest reliability of the digital inclinometer app when recording five repeated trials. The knee proprioception pre and post an intervening activity demonstrated poor correlation measurements separated by a delay in time, leading to some hesitation in trusting that one's aJPS remains the same from visit to visit. However, it should be noted that in this knee dataset, in between tests participants performed several activities including single-leg landings and isokinetic strength testing. Therefore, we do not know at present whether the differences seen between knee aJPS sessions were due to natural variation over time or due to the intervening activities altering one's JPS. A recent study looking into effects on proprioception immediately following exercise showed a decrease in proprioceptive ability directly after completion of the exercise and returned

to pre-exercise levels after 72 hours of passive recovery [23]. This may suggest that proprioception can be affected due to intervening activities and exercises, supporting our results of poor correlation of knee proprioception before and after the activities.

The knee proprioception reliability results are supported by other literature, such as a study performed to assess the reliability of a custom-built web application running on a smartphone, with an isokinetic dynamometer used as the main control [24]. In this study, Ravi concluded that the standard deviations obtained were less than 0.5 degrees for the knee angle measurements on the web-application against the isokinetic dynamometer. The statistical analyses demonstrated no significant difference between the web application and the isokinetic dynamometer. These data together demonstrate the high degree of reliability and validity of the web-based measurement of knee joint angle, closely aligned to measurements obtained on the isokinetic dynamometer [24]. The current study builds upon these results by showing comparable reliability values when measuring knee joint position sense error, as well as showing little difference between data collected before and after an intervening activity.

Ankle proprioception

Results of the ankle aJPS testing showed poor test-retest reliability of the digital inclinometer app when recording only five repeated trials, but good reliability when 10 trials were collected. This result suggests that ankle proprioception is more variable than the knee and hip, but future research is necessary to test this hypothesis. Care should be taken in the future to ensure enough trials are collected for a reliable ankle proprioception assessment.

These results build upon data collected and analyzed in a prior study that aimed to test the reliability of smartphone goniometry in the ankle joint by comparing it with a universal goniometer and to assess inter-rater and intra-rater reliability for the smartphone goniometer record application [25]. In this study, Alawna et al. found that, for measuring ankle dorsiflexion range of motion (ROM), both instruments showed excellent interrater reliability. Intra-rater reliability was excellent in both instruments

in ankle dorsiflexion, and as for measuring ankle plantarflexion, both instruments showed excellent interrater and intra-rater reliability. Similarly to the hip proprioception literature previously mentioned, these data show great reliability when comparing smartphone and universal goniometry joint angle measurements. The current study extends these results by comparing ankle joint position sense error values and by determining the number of trials that must be collected to establish good reliability.

Limitations

It is important to highlight that the data described in this paper are made up of three separately collected datasets. Each dataset, for the hip, knee, and ankle aJPS, was individually collected at different times, by different testers, in different environments, and with different participants. In the future, it may be more appropriate to collect a single dataset with the same sets of testers and participants, allowing for more direct comparison of the joint measurements to one another.

Some important considerations when using digital inclinometer apps in the testing protocols described above, or similar, include: ensuring the sensor is attached in the correct orientation; ensuring the sensor is attached securely to each participant, so as to minimize any extra noise or error from it shifting its position during dynamic tasks; and ensuring that whatever "learned position" remains consistent across participants and trials, whether that be through the use of a device or environmental cue. In addition, a specific digital inclinometer app was used for this study, and no comparison was made to other available digital inclinometers. This limitation is considered to be minor, because both the inertial sensors and data processing to estimate device angle represent common standards across the many available digital inclinometers such as the EasyAngle® digital goniometer (Meloq AB, Stockholm, Sweden) or the RST Instruments digital inclinometer (RST Instruments Ltd, Maple Ridge, Canada).

Future research should include a more robust device to cue the "learned position", taking care to ensure that the design remains fairly simple to translate to a clinical setting, so that the participant does not change position with respect to the apparatus throughout the trials for better and more consistent data

collection. In addition, more diversity of the population in age, joint pathologies or neurological conditions and a higher number of participants would improve the generalizability of the results.

Conclusion

A digital inclinometer app achieved good intra-session reliability at measuring joint position sense in the hip, knee, and ankle with an appropriate number of trials and a trimmed-average approach. These results should be generalizable across the many devices on the market that can be used similarly to the digital inclinometer app system in this study. This tool and methodology can be considered for use in both clinical and research environments to measure proprioception and proprioceptive deficits.

Acknowledgments

The authors would like to thank Al Caserta, Pierce Fonseca, Atticus Jordan, Nelson Glover, Andrea Wanamaker, Kenechukwu Okoye, Michael McNally, Kimberly Bigelow, Paige Ingram, Sarah Hollis, Amanda Delaney, Jack Schultz, Jennifer Perry, Chelsea Jentsch, Cruz Finnicum, Cody Ballay, and Orion Swanson for their time and hard work involved in the collection of the data reported in this paper. We would also like to thank the American Society of Biomechanics for allowing and supporting our data collection during the 2017 annual meeting in Boulder, Colorado.

References

1. Han J, Waddington G, Adams R, Anson J, Liu Y. Assessing proprioception: A critical review of methods. J Sport Health Sci. 2016;5: 80–90. doi:10.1016/j.jshs.2014.10.004

2. Röijezon U, Clark NC, Treleaven J. Proprioception in musculoskeletal rehabilitation. Part 1: Basic science and principles of assessment and clinical interventions. Man Ther. 2015;20: 368–377. doi:10.1016/j.math.2015.01.008

3. Clark NC, Röijezon U, Treleaven J. Proprioception in musculoskeletal rehabilitation. Part 2: Clinical assessment and intervention. Man Ther. 2015;20: 378–387. doi:10.1016/j.math.2015.01.009

4. Wang H, Ji Z, Jiang G, Liu W, Jiao X. Correlation among proprioception, muscle strength, and balance. J Phys Ther Sci. 2016;28: 3468–3472. doi:10.1589/jpts.28.3468

5. Kim H-J, Lee J-H, Lee D-H. Proprioception in Patients With Anterior Cruciate Ligament Tears: A Meta-analysis Comparing Injured and Uninjured Limbs. Am J Sports Med. 2017;45: 2916–2922. doi:10.1177/0363546516682231

6. Chaput M, Onate JA, Simon JE, Criss CR, Jamison S, McNally M, et al. Visual cognition associated with knee proprioception, time to stability, and sensory integration neural activity after ACL reconstruction. Journal of Orthopaedic Research. 2022;40: 95–104. doi:10.1002/jor.25014

7. Knoop J, Steultjens MPM, van der Leeden M, van der Esch M, Thorstensson CA, Roorda LD, et al. Proprioception in knee osteoarthritis: a narrative review. Osteoarthritis Cartilage. 2011;19: 381–388. doi:10.1016/j.joca.2011.01.003

8. Elangovan N, Herrmann A, Konczak J. Assessing Proprioceptive Function: Evaluating Joint Position Matching Methods Against Psychophysical Thresholds. Phys Ther. 2014;94: 553–561. doi:10.2522/ptj.20130103

9. Felson DT, Gross KD, Nevitt MC, Yang M, Lane NE, Torner JC, et al. The effects of impaired joint position sense on the development and progression of pain and structural damage in knee osteoarthritis. Arthritis Rheum. 2009;61: 1070–1076. doi:10.1002/art.24606

10. Cooper R, Taylor N, Feller J. A Systematic Review of the Effect Of Proprioceptive and Balance Exercises on People With an Injured Or Reconstructed Anterior Cruciate Ligament. Research in sports medicine (Print). 2005;13: 163–78. doi:10.1080/15438620590956197

11. Hillier S, Immink M, Thewlis D. Assessing Proprioception: A Systematic Review of Possibilities. Neurorehabil Neural Repair. 2015;29: 933–949. doi:10.1177/1545968315573055

12. Kiran D, Carlson M, Medrano D, Smith DR. Correlation of three different knee joint position sense measures. Phys Ther Sport. 2010;11: 81–85. doi:10.1016/j.ptsp.2010.06.002

13. Suner-Keklik S, Cobanoglu-Seven G, Kafa N, Ugurlu M, Guzel NA. The Validity and Reliability of Knee Proprioception Measurement Performed With Inclinometer in Different Positions. J Sport Rehabil. 2017;26. doi:10.1123/jsr.2017-0010

14. Ko S-U, Simonsick E, Deshpande N, Ferrucci L. Sex-specific age associations of ankle proprioception test performance in older adults: results from the Baltimore Longitudinal Study of Aging. Age and Ageing. 2015;44: 485–490. doi:10.1093/ageing/afv005

15. Mourcou Q, Fleury A, Diot B, Franco C, Vuillerme N. Mobile Phone-Based Joint Angle Measurement for Functional Assessment and Rehabilitation of Proprioception. Biomed Res Int. 2015;2015: 328142. doi:10.1155/2015/328142

16. Busch A, Bangerter C, Mayer F, Baur H. Reliability of the active knee joint position sense test and influence of limb dominance and sex. Sci Rep. 2023;13: 152. doi:10.1038/s41598-022-26932-2

17. Dover G, Powers ME. Reliability of Joint Position Sense and Force-Reproduction Measures During Internal and External Rotation of the Shoulder. J Athl Train. 2003;38: 304–310.

18. Kunritt J, Lekskulchai R, Wattananon P. How Many Trials are Needed to Assess Ankle Joint Proprioception in Children with Chronic Ankle Instability? Trends in Sciences. 2021;18: 25.

19. Chaudhari AMW, McKenzie CS, Pan X, Oñate JA. Lumbopelvic control and days missed due to injury in professional baseball pitchers. Am J Sports Med. 2014;42: 2734–2740. doi:10.1177/0363546514545861

20. Core Motion. In: Apple Developer Documentation [Internet]. [cited 29 Sep 2023]. Available: https://developer.apple.com/documentation/coremotion

21. Portney LG, Watkins MP. Foundations of Clinical Research: Applications to Practice. Pearson/Prentice Hall; 2009.

22. Cairo University, Faculty of Physical Therapy, Department of Biomechanics, Giza, Egypt, Mohammad WS, Elattar FF, Cairo University, Faculty of Physical Therapy, Supervisor of Orthopedic Outpatient Clinic, Giza, Egypt, Elsais WM, Isra University, College of Allied Medical Sciences, Department of Physical Therapy, Amman, Jordan, et al. Validity and Reliability of a Smartphone and Digital Inclinometer in Measuring the Lower Extremity Joints Range of Motion. Monten J Sports Sci Med. 2021;10: 47–52. doi:10.26773/mjssm.210907

23. Naderi A, Rezvani MH, Degens H. Foam Rolling and Muscle and Joint Proprioception After Exercise-Induced Muscle Damage. J Athl Train. 2020;55: 58–64. doi:10.4085/1062-6050-459-18

24. Ravi B, Kapoor M, Player D. Feasibility and reliability of a web-based smartphone application for joint position measurement. J Rehabil Med. 2021;53: jrm00188. doi:10.2340/16501977-2780

25. Alawna MA, Unver BH, Yuksel EO. The Reliability of a Smartphone Goniometer Application Compared With a Traditional Goniometer for Measuring Ankle Joint Range of Motion. J Am Podiatr Med Assoc. 2019;109: 22–29. doi:10.7547/16-128

Figure 2

Click here to access/download;Figure;Fig2.tif

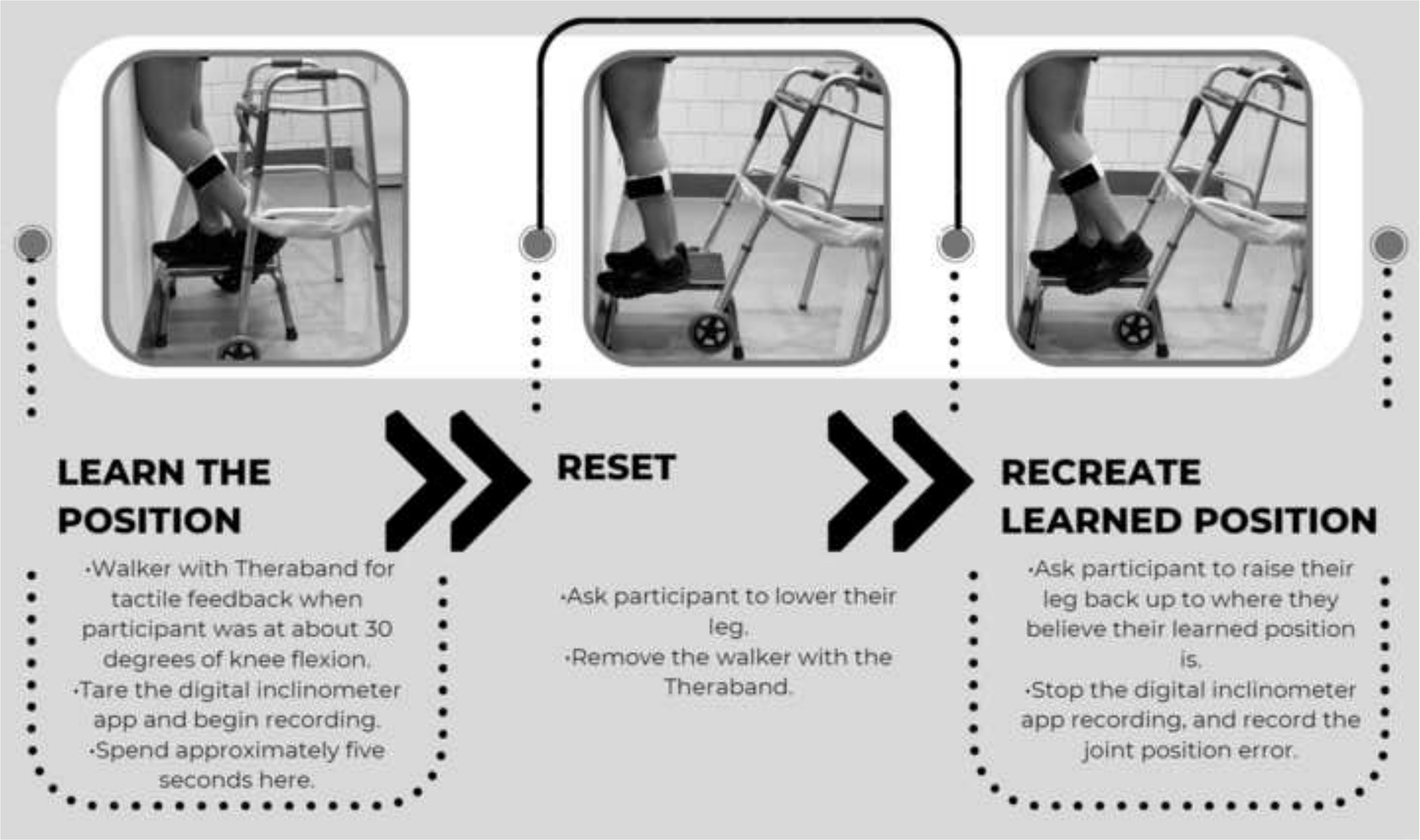

Figure 3

Click here to access/download;Figure;Fig3.tif

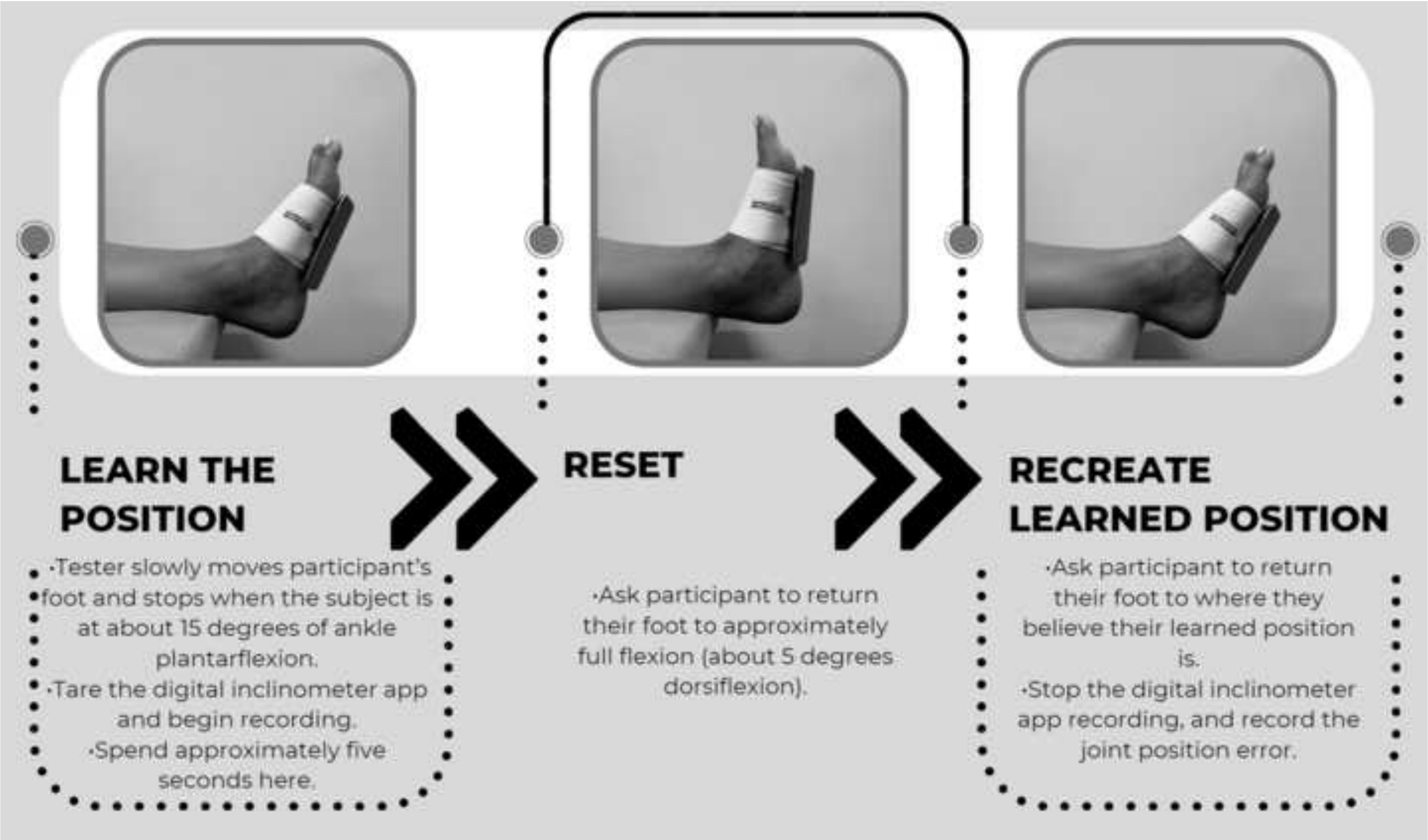

Figure 1

Click here to access/download;Figure;Fig1.tif

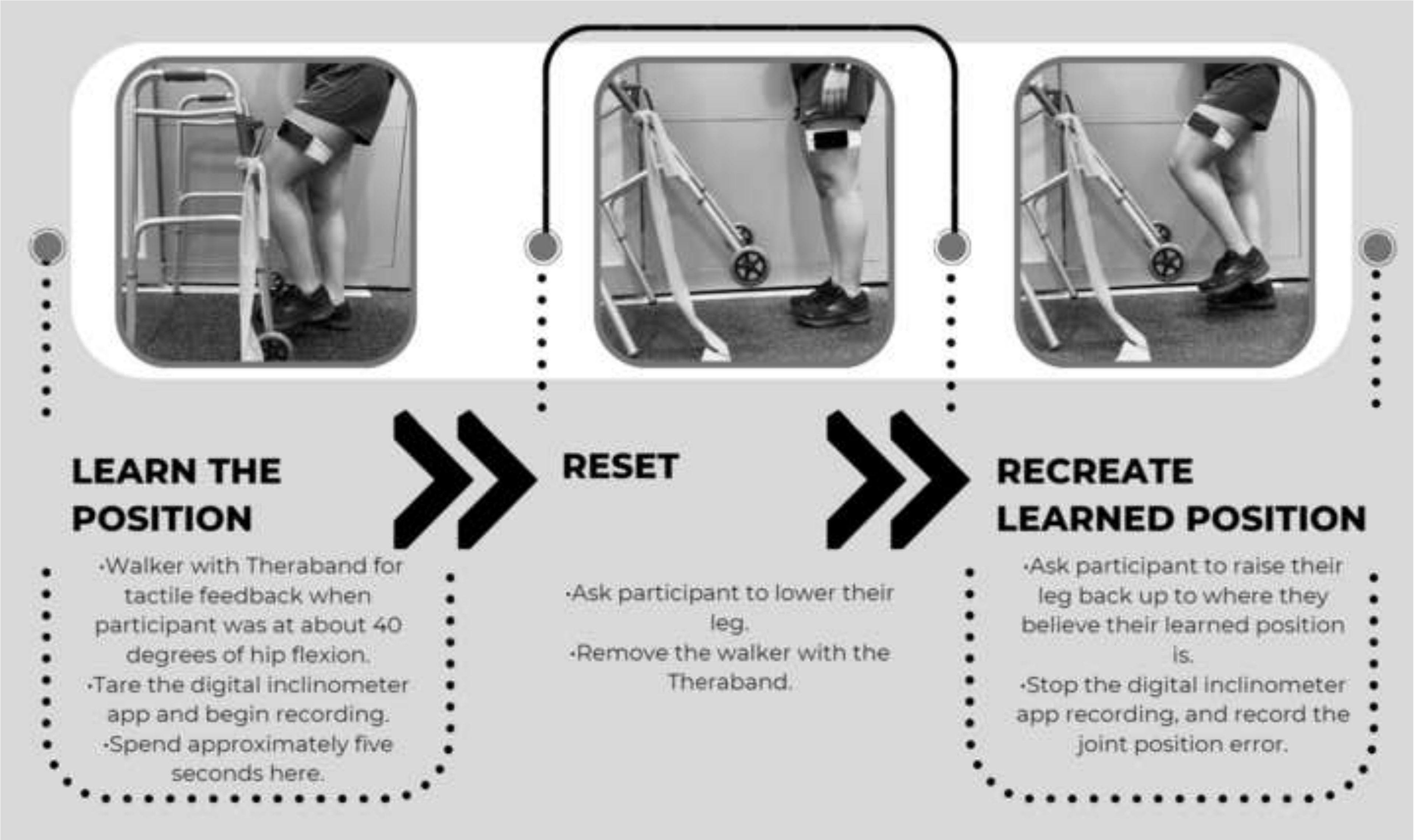

Click here to access/download
Supporting Information
Clean Raw Data - Assessing.xlsx
