# Supplementary material for: Assessing the reliability of a digital inclinometer app for measuring hip, knee, and ankle proprioception
Source: PLoS One. 2024 Sep 23;19(9):e0308737. doi: 10.1371/journal.pone.0308737 (PMC11419353; doi:10.1371/journal.pone.0308737)
Supplement: Attachment — Submitted filename: Response to Reviewers.docx [file pone.0308737.s003.docx]

Reviewer comments will be in *italics* and author responses will be located directly below in **bold**.

**Reviewer #1:**

*Abstract: It is recommended to include the 95% confidence intervals (CI) for the ICC values.*

**Thank you for your comment. We have included the 95% confidence intervals for each of the ICC values in an updated table (Table 1), as well as mentioned them within the text in brackets when reporting specific ICC values.**

*Methods: To strengthen the methodology, consider providing more details on how the sample size was calculated. This will enhance the transparency and generalizability of the study.*

**Thank you for your comment. The following text was added, and edited, to each joint’s study participant section for further explanation, accordingly.**

**Hip: “A convenience sample of forty-two participants were recruited and tested between the months of March and April 2017 at the Ohio State University. A total of 17 males and 25 females were included in this portion of the study, with ages ranging from 21-31 years old. Hip proprioception data were originally collected as part of an educational assignment for a course.” (Page 5, Lines 109-111)**

**Knee: “This sample size is larger than the projected minimum of twenty-six participants, that provides 0.8 power to detect an ICC(2,k) = 0.8 compared to a null hypothesis of ICC(2,k) = 0.6 that will be used to demonstrate the test-retest reliability of the iPod app assessment.” (Page 6, Lines 118-121)**

**Ankle: “This was the maximum number of participants able to have their data recorded within the time period allotted for data collection during the conference.” (Page 6, Lines 127-129)** *Methods: It would be helpful to explain the rationale behind choosing ICC (2,1) and ICC (2,k) as the specific intraclass correlation coefficients. Knowing the justification for these selections will improve the clarity of the research design.*
**Thank you for your comment. We have added the following text to address this: “The intraclass correlation coefficient (ICC) and associated 95% confidence interval (95% CI) were calculated between aJPS error values because they are commonly used to assess consistency across, or within, a dataset. [22] The ICC numerical values themselves should be identical whether one uses ICC (2,k) or ICC (3,k). The importance of the distinction between the two lies in the interpretation of the data. ICC (2,k) assumes random-effects, which makes its interpretation generalizable across populations, whereas ICC (3,k) assumes fixed-effects. [23] Therefore, ICC (2,k) is reported here to determine if the aJPS measurements are generalizable across a greater participant population, as well as among other raters, or testers. Additionally, ICC (2,1) is reported to determine the data’s validity and reliability if only a single measure is recorded. This is applicable in the current article, as it is suggested that this assessment tool may be used in clinical settings, where efficiency is important.” (Page 9, Lines 190-199)**

*Methods (Statistical analyses): Including standard error of measurement data would be beneficial. This additional information will provide a more comprehensive understanding of the measurement reliability.*
**Thank you for your comment. We have included the standard error of measurement values in an updated table (Table 1), located below.**

| ***Joint*** | ***Number of Trials*** | ***ICC Test*** | ***ICC Value*** | ***95% Confidence Interval*** | ***Standard Error (deg.)*** |
| --- | --- | --- | --- | --- | --- |
| **Hip** | **5 trials** | **ICC (2,1)** | 0.388 | [0.288 - 0.499] | 1.6 |
|  |  | **ICC (2,k)** | 0.760 | [0.669 - 0.833] |  |
| **Hip** | **Middle 3 of 5 trials** | **ICC (2,k)** | 0.849 | [0.783 – 0.897] | 1.0 |
| **Hip** | **Inter-limb “trimmed average”** | **ICC (2,1)** | 0.508 | [0.245 – 0.701] | 1.1 |
| **Knee** | **5 trials** | **ICC (2,1)** | 0.276 | [0.165 – 0.410] | 1.2 |
|  |  | **ICC (2,k)** | 0.656 | [0.497 – 0.777] |  |
| **Knee** | **Middle 3 of 5 trials** | **ICC (2,k)** | 0.837 | [0.750 – 0.897] | 0.7 |
| **Knee** | **Pre & post time delay “trimmed average”** | **ICC (2,1)** | 0.429 | [0.083 – 0.682] | 0.7 |
| **Ankle** | **10 trials** | **ICC (2,1)** | 0.176 | [0.099 – 0.289] | 2.3 |
|  |  | **ICC (2,k)** | 0.682 | [0.524 – 0.803] |  |
| **Ankle** | **First 5 trials** | **ICC (2,k)** | 0.481 | [0.200 – 0.685] | 2.3 |
| **Ankle** | **Middle 3 of first 5 trials** | **ICC (2,k)** | 0.585 | [0.324 – 0.757] | 1.4 |
| **Ankle** | **Middle 8 of 10 trials** | **ICC (2,k)** | 0.785 | [0.539 – 0.893] | 0.9 |

*Results: The results section would benefit from reporting the mean difference between the readings along with their corresponding significance levels. This will allow readers to better interpret the statistical significance of the findings.*
**Thank you for your comment. We have included the standard error of measurement values in an updated table (Table 1). We have included an example Bland-Altman plot and histogram for ankle aJPS, illustrating low systematic bias of over- or under-estimation of aJPS error (Figures 7 & 8). We have also included plots comparing each participant’s standard deviation vs. mean aJPS error measurements in the Results section (Figures 4-6).**

*Results: To improve the clarity and precision of the findings, it's recommended to include the 95% CI for the ICC values.*

**Thank you for your comment. We have included the 95% confidence intervals for each of the ICC values in an updated table (Table 1), as well as mentioned them within the text in brackets when reporting specific ICC values.**

*Results: Presenting the data in a well-organized table would enhance readability and facilitate easier comparison between different groups or variables.*

**Thank you for your comment. We have created an updated table. (Table 1)**

*Results: I recommend using Bland-Altman plots to visualize potential systematic bias between the readings.*

**Thank you for your comment. We have included an example Bland-Altman plot and histogram for ankle aJPS, illustrating low systematic bias of over- or under-estimation of aJPS error (Figures 7 & 8). We have also included plots comparing each participant’s standard deviation vs. mean aJPS error measurements in the Results section (Figures 4-6). We have also added the following text related to our interpretation of these figures:**

**Hip: “The plot shows that with increasing aJPS error at the hip, variance increases (Fig 4).” (Page 11, Lines 225-226)**

**Knee: “The plot shows that with increasing aJPS error at the knee, variance increases (Fig 5).” (Page 12, Lines 240-241)**

**Ankle: “The plot shows that with increasing aJPS error at the ankle, variance increases (Fig 6).” (Page 12, Lines 254-255)**

**“Additionally, directional systematic bias during trials was negligibly low, illustrated by the example Bland-Altman plot and histogram for ankle aJPS error (Fig 7 & 8). The histogram below shows that the distribution of the raw ankle aJPS error data points are centered around zero without any noticeable skew (Fig 8). Furthermore, the Bland-Altman plot and the plots of standard deviation of error shown in Figures 4-6 demonstrate that those with larger mean aJPS error also generally experience greater variability across individual measurements” (Page 12-13, Lines 260-265)**

Reviewer #2: *Kindly specify the number of trials you have undertaken for each joint specifically.*

**Thank you for your comment. Specific trial numbers have been added to the text when referencing trials. (Page 2, Lines 23-24) & (Page 7, Lines 137-138)**

Reviewer #3:

*I think you could elaborate the statistical analysis for better results. Why did not you use SEM, MDC or even Cronbach's alpha? It seems the possible error of measurement should be calculated to make sure that the measurements are not because of errors.*
**Thank you for your comment. We have included the standard error of measurement values in an updated table (Table 1). We have also added the following text detailing the standard error of measurement calculation: “Additionally, the standard error of measurement (SEM) was calculated for the aJPS error values at each joint. The SEM was calculated by taking the square root of the residual mean square error value, given by the SPSS software, for each separate reliability analysis, accordingly [24].” (Page 10, Lines 205-208)**

**We have also added the following text to address the remainder of your comment: “The intraclass correlation coefficient (ICC) and associated 95% confidence interval (95% CI) were calculated between aJPS error values because they are commonly used to assess consistency across, or within, a dataset. [22] The ICC numerical values themselves should be identical whether one uses ICC (2,k) or ICC (3,k). The importance of the distinction between the two lies in the interpretation of the data. ICC (2,k) assumes random-effects, which makes its interpretation generalizable across populations, whereas ICC (3,k) assumes fixed-effects. [23] Therefore, ICC (2,k) is reported here to determine if the aJPS measurements are generalizable across a greater participant population, as well as among other raters, or testers. Additionally, ICC (2,1) is reported to determine the data’s validity and reliability if only a single measure is recorded. This is applicable in the current article, as it is suggested that this assessment tool may be used in clinical settings, where efficiency is important.” (Page 9, Lines 190-199)**

*For using new apps or even new devices, finding validity of the device is necessary. However, How did you know the app is valid? have you checked it with a gold standard?*

**Thank you for your comment. We have added, and edited, the following text to address this: “This article reports the reliability and validity of a digital inclinometer app that has been previously validated for quantifying pelvic motion. When compared with motion capture, considered a “gold standard” in this previous study, the digital inclinometer app was found to have good reliability for measurement of pelvic motion [19].” (Page 5, Lines 94-97)**

**We have also added a reference for the application code and details. (Page 7, Line 144)**
